# Highlights in USP7 inhibitors for cancer treatment

**DOI:** 10.3389/fchem.2022.1005727

**Published:** 2022-09-15

**Authors:** Rita I. Oliveira, Romina A. Guedes, Jorge A. R. Salvador

**Affiliations:** ^1^ Laboratory of Pharmaceutical Chemistry, Faculty of Pharmacy, University of Coimbra, Coimbra, Portugal; ^2^ Center for Neuroscience and Cell Biology, University of Coimbra, Coimbra, Portugal

**Keywords:** ubiquitin proteasome system, deubiquitinases, USP7, USP7 inhibitors, small molecules, cancer treatment

## Abstract

Ubiquitin-specific protease 7 (USP7) is a member of one of the most largely studied families of deubiquitylating enzymes. It plays a key role modulating the levels of multiple proteins, including tumor suppressors, transcription factors, epigenetic modulators, DNA repair proteins, and regulators of the immune response. The abnormal expression of USP7 is found in various malignant tumors and a high expression signature generally indicates poor tumor prognosis. This suggests USP7 as a promising prognostic and druggable target for cancer therapy. Nonetheless, no approved drugs targeting USP7 have already entered clinical trials. Therefore, the development of potent and selective USP7 inhibitors still requires intensive research and development efforts before the pre-clinical benefits translate into the clinic. This mini review systematically summarizes the role of USP7 as a drug target for cancer therapeutics, as well as the scaffolds, activities, and binding modes of some of the most representative small molecule USP7 inhibitors reported in the scientific literature. To wind up, development challenges and potential combination therapies using USP7 inhibitors for less tractable tumors are also disclosed.

## Introduction

**FIGURE 1:**
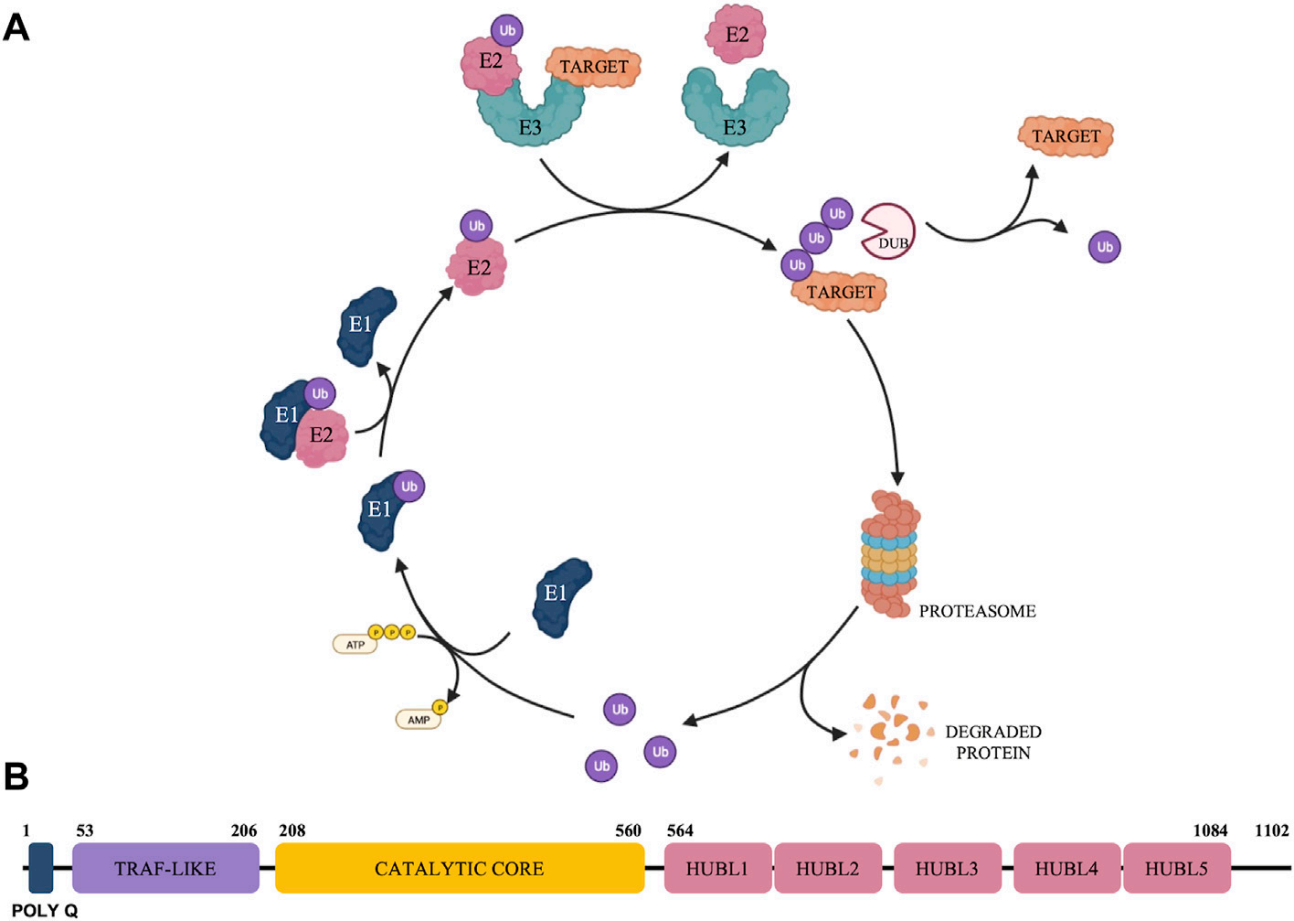
Schematic representation of the Ubiquitin Proteasome System **(A)** and overview of USP7 structure **(B)**.

The ubiquitin-proteasome system (UPS) (represented in Figure 1A) has emerged as a crucial regulator in a broad spectrum of biological functions and diseases including cancer (Selvaraju et al., 2015). The UPS is a nonlysosomal intracellular protein degradation pathway which comprises a group of enzymes that tag proteins for destruction with the small-molecule ubiquitin (Ub) and the multi-subunit proteolytic complex—the 26S proteasome (Hershko, 2005; D’Arcy et al., 2015). Protein ubiquitination is a dynamic, tightly controlled, and reversible post-translational modification with the 76 amino-acid protein Ub that is involved in the regulation of most cellular processes (Yau and Rape, 2016; Clague et al., 2019). The ubiquitination process is mediated by an enzymatic cascade that depends on the continuous activity of three catalyzing enzymes, E1 (ubiquitin-activating enzyme), E2 (ubiquitin-conjugating enzymes) and E3 (ubiquitin-protein ligases) (Fang and Weissman, 2004). Deubiquitinases (DUBs) exert their functions by reversing the monoubiquitination or polyubiquitination of target protein (Mevissen and Komander, 2017) and are associated with the 26S proteasome to remove Ub chains before the degradation of the substrate proteins, being responsible to maintain the dynamic state of the cellular ubiquitome (Clague et al., 2019).

There are approximately 100 DUBs divided into two main classes according to their enzymatic cleavage mechanism: cysteine proteases and zinc metalloproteases (Nijman et al., 2005; Schauer et al., 2020b). Cysteine proteases can be further divided into six families based on phylogenetic relationships’, namely ubiquitin-specific proteases (USPs), ovarian tumor proteases (OTUs), Machado−Josephin domain proteases (MJDs), ubiquitin C-terminal hydroxylases (UCHs), motif interacting with ubiquitin-containing novel DUB family proteases (MINDYs), and zinc finger-containing ubiquitin peptidase 1 (ZUP1). Jab1/Mov34/Mpr1 Pad1 N-terminal+ domain proteases (JAMMs) are the only family reported so far with a zinc metalloprotease active site fold. Among these, USPs are the largest DUB family with ∼60 proteases in humans and USP7 is one of the most intensively studied due to its pivotal role in cancer progression (Schauer et al., 2020b). USP7, also known as herpesvirus-associated ubiquitin-specific protease, is involved in modulating the stability of several key proteins, including tumor suppressors (Li et al., 2002; Song et al., 2008), transcription factors (van der Horst et al., 2006), epigenetic modulators (Du et al., 2010; Wang et al., 2016a; Wang et al., 2016b), proteins involved in DNA damage response (Zhu et al., 2015; Su et al., 2018) and regulators of immune response (van Loosdregt et al., 2013).

Interest in USP7 has been intensified when it was implicated in modulating the degradation of the tumor suppressor p53 through the stabilization of its major E3 ligase, murine double minute 2 (MDM2), causing dysregulation of the MDM2-p53 pathway in malignant tumors. Notably, USP7 overexpression has been demonstrated to stabilize MDM2 and destabilize p53, promoting p53 proteasomal degradation and diminishing p53 tumor suppressor downstream networks (Li et al., 2004). Therefore, the development of potent and selective small molecule inhibitors of USP7 has the potential to be a promising treatment for cancers and other disorders and has become a highly sought-after goal.

In this review, we will provide a summary overview of the recent advances in the development of USP7 inhibitors for cancer treatment.

## Structural characteristics of ubiquitin-specific protease 7

USP7 is a multi-domain DUB with catalytic and non-catalytic domains which participates in the tight regulation of both substrate recognition and deubiquitinase activity (Rougé et al., 2016). USP7 has 1,102 amino acid residues that can be further subdivided into the following domains: a highly conserved amino-terminal poly-glutamine (poly Q) stretch located at the very N-terminus, an N-terminal tumor necrosis factor receptor-associated factor (TRAF)-like domain, a central catalytic domain, 5 C-terminal ubiquitin-like domains (HUBL1-5) and 19 C-terminal amino acid residues (Bhattacharya et al., 2018) (Figure 1B).

The catalytic domain of all USPs show a characteristic papain-like fold with an architecture that resembles an open hand with fingers, palm, and thumb subdomains (Turcu et al., 2009). USP7 exists in a catalytically incompetent apoenzyme form characterized by a highly conserved catalytic triad located between the palm and thumb subdomains. The amino acids from the triad include Cysteine 223 (Cys223), Histidine 464 and Aspartic acid 481 (Wertz and Murray, 2019), which are misaligned and postulated to rearrange into a productive conformation only upon Ub binding, yielding the catalytic triad residues to come into proximity for efficient ubiquitin catalysis (Hu et al., 2002).

## Ubiquitin-specific protease 7 as a drug target for cancer therapeutics

USP7 is emerging as a promising pharmacological target for interference with the UPS in several human cancers. Studies have shown the role of USP7 in the regulation of numerous cancer-related pathways and proven that high levels of USP7 are directly correlated with tumor progression in numerous cancers including breast cancer (Wang et al., 2016b), ovarian cancer (Qin et al., 2016), prostate cancer (Song et al., 2008), cervical cancer (Su et al., 2018) and colorectal cancer (An et al., 2017), among others.

In breast cancer cells, USP7 is able to physically interact with estrogen receptor α (ERα) and the histone demethylase plant homeodomain finger–containing protein 8 (PHF8) to promote their stabilization and lead to the upregulation of a group of genes that are critical for cell growth and proliferation. Therefore, USP7 is able to promote breast carcinogenesis through the USP7/PHF8/cyclin A2 axis (Wang et al., 2016b; Xia et al., 2019). Interestingly, USP7 was the first DUB identified to mediate ERα expression and USP7 protein expression was positively correlated with that of ERα (Xia et al., 2019). Moreover, USP7 expression levels were linked with geminin levels in a collection of invasive breast cancers and related to genome instability, DNA replication changes and aneuploidy (Hernández-Pérez et al., 2017).

In prostate cancer, USP7 can deubiquitinate and stabilize the androgen receptor in an androgen dependent manner, promoting the androgen receptor binding to chromatin for the transcription of target genes related with cell growth and proliferation (Morra et al., 2017). Furthermore, USP7 overexpression reduces phosphatase and tensin homolog (PTEN) monoubiquitination and leads to PTEN nuclear exclusion, which is associated with a more aggressive phenotype (Song et al., 2008).

In cervical cancer, USP7 is reported to physically associate with the MRE11-RAD50-NBS1 (MRN)—mediator of DNA damage checkpoint protein 1 (MDC1) complex, enabling DNA repair through the stabilization of MDC1. This will maintain the MRN-MDC1 complex around DNA double strand breaks, conferring resistance of cervical cancer cells to genotoxic insults, therefore promoting cervical carcinogenesis (Su et al., 2018).

In colorectal cancer, USP7 can participate in the aberrant activation of the Wnt/β-catenin signaling through stabilization of the key transcriptional modulator β-catenin (An et al., 2017). Studies report this enhanced signaling to be promoted by a complex formed between the E3 ligase Ring Finger Protein 220 (RNF220) and USP7 in which RNF220 operates as an adaptor that bridges USP7 and β-catenin (Ma et al., 2014).

Collectively, the results from these studies support the pursuit of USP7 as a promising and attractive drug target for cancer therapeutics.

## Challenges in ubiquitin-specific protease 7 inhibitor development

The development of effective USP7 inhibitors (USP7i) faces many challenges. First, the selectivity against USP7 over other DUBs must be wisely taken into consideration to prevent cross-inhibition effects. This has been challenging partially due to the highly conserved structural features of the catalytic domain across the class (Zhang et al., 2017). Additionally, it is worth noting that no individual DUB selectivity platform has a comprehensive coverage of the 100 reported DUBs, further requiring complementary selectivity assays (Schauer et al., 2020b). Additional challenges to overcome include the weak micromolar potency presented by most USP7i. Only a small set are in the low nanomolar range. Therefore, finding new allosteric binding sites within USP7 to design novel inhibitors may be a promising way to improve potency and also selectivity due to their low homology against other DUBs (Lamberto et al., 2017). Furthermore, there is a need for faster and more precise screening methods for USP7i. Traditional methods include Ub-Rhodamine and Ub-7-amido-4-methylcoumarin (Ub-AMC) that despite widely used and cost-effective are not sufficiently accurate if the test compounds are fluorescent. Moreover, modern methods, including surface plasmon resonance, are costly and have low efficiency in the large scale. Therefore, new screening methods that cautiously balance accuracy and efficiency will certainly benefit the development of USP7i (Li and Liu, 2020; Li et al., 2022).

Over the last decade, the searching for USP7i has witnessed intensive efforts. Even though there are no relevant inhibitors that have entered clinical trials so far, the further development and clinical testing of USP7i emerges as a highly sought-after and likely valuable new approach for the treatment of various cancers. Herein are described some of the most promising USP7i and relevant findings, subclassified based on the core functional groups that interacted with USP7 (Schauer et al., 2020b; Pereira et al., 2022).

### Thiophene derivatives

Small molecules derivatives of the thiophene scaffold have been reported as irreversible USP7i. These molecules target the catalytic cleft of USP7, forming a covalent adduct with the active site Cys223. The presence of a doubly deactivated, highly electron-deficient thiophene ring indicates that these molecules may react directly with nucleophilic residues, such as the catalytic Cys223, therefore leading to covalently modified adducts. Modification of the Cys223 thiol group will irreversibly inhibit the enzymatic activity of USP7 by averting the formation of anionic sulfur and its subsequent nucleophilic attack on the C-terminal carbonyl carbon of Ub (Pozhidaeva et al., 2017). The most representative scaffolds include P5091 (Chauhan et al., 2009) (Table 1), P22077 (Fan et al., 2013), P50429 (Weinstock et al., 2012) and P217564 (Wang et al., 2017a). Despite the USP7 inhibitory activity, these molecules have been shown to be dual inhibitors of USP7 and its closest homolog USP47, inhibiting both enzymes with similar potencies (Chauhan et al., 2009; Weinstock et al., 2012; Fan et al., 2013).

**TABLE 1:** Representative structures of the most potent USP7 inhibitors scaffolds.

Scaffold	*In vitro* functional activity	Cancer models targeted *in vitro*	Cancer models targeted *in vivo*	References
Thiophene derivatives P5091 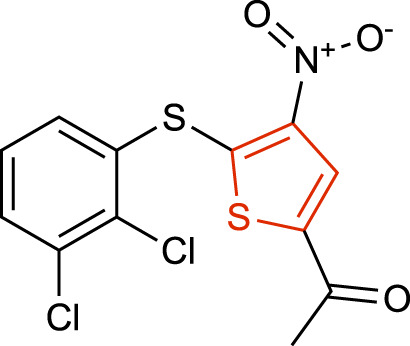	IC_50_ = 4.2 μM	MM cells, Colorectal cancer cells, Prostate cancer cells (hormone-sensitive and castration resistant), Ovarian cancer cells, Chronic lymphocytic leukemia cells, MM purified patient cells (IC_50_ ≤ 3 μM), Lung neuroendocrine cancer cells	MM, colorectal cancer, and glioblastoma xenograft models	Chauhan et al. (2012); An et al. (2017); Carrà et al. (2017); Malapelle et al. (2017); Morra et al. (2017); Wang et al. (2017b)
Amidotetrahydroacridine derivatives HBX19,818 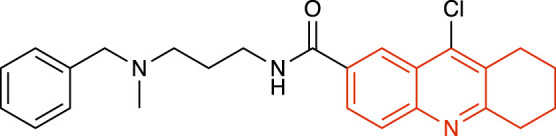	IC_50_ = 28.1 μM	Colorectal cancer cells (IC_50_ ∼ 2 μM), Chronic lymphocytic leukemia cells	Not found	Reverdy et al. (2012); Agathanggelou et al. (2017)
Quinazolin-4-one derivatives C19 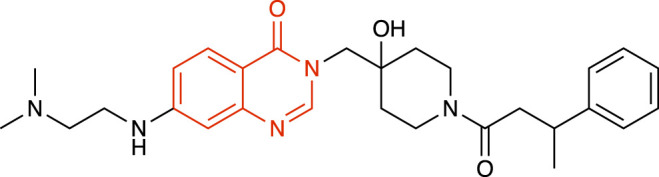	IC_50_ = 0.595 μM	Gastric cancer cells	Not found	Li et al. (2021)
Cyanopyrrolidine derivatives Compound 11 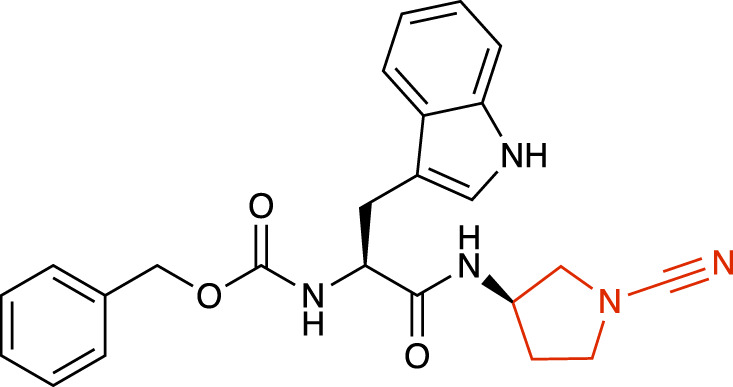	IC_50_ = 12.1 μM	Not found	Not found	Bashore et al. (2020)
Sesquiterpene lactone derivatives Parthenolide 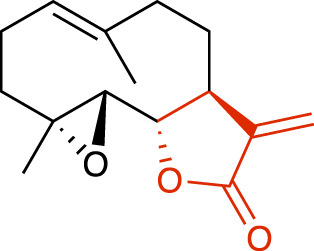	IC_50_ = 6.58 μM (Ub-AMC assay), IC_50_ = 15.42 μM (Ub-rhodamine assay)	Colorectal cancer cells	Colorectal cancer xenograft models	Li et al. (2020a)
2-amino-4-ethylpyridin derivatives GNE-6640 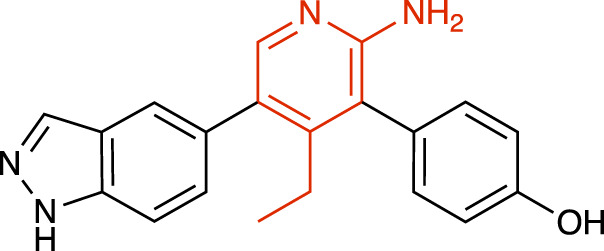	IC_50_ = 0.75 μM	Colorectal cancer cells, Acute myeloid leukemia cells, Osteosarcoma cells	Not found	Di Lello et al. (2017); Kategaya et al. (2017)
4-hydroxypiperidine derivatives XL188 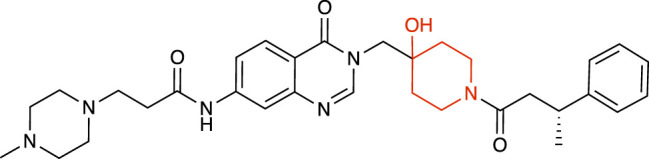	IC_50_ = 90 nM	MM cells, Breast cancer cells, Ewing sarcoma cells	Not found	Lamberto et al. (2017); Stolte et al. (2018)
4-hydroxypiperidine derivatives XL177A 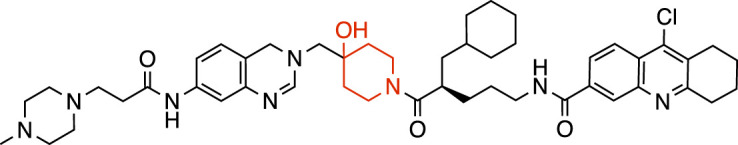	IC_50_ = 0.34 nM	Breast cancer cells	Not found	Schauer et al. (2020a)
4-hydroxypiperidine derivatives ALM34 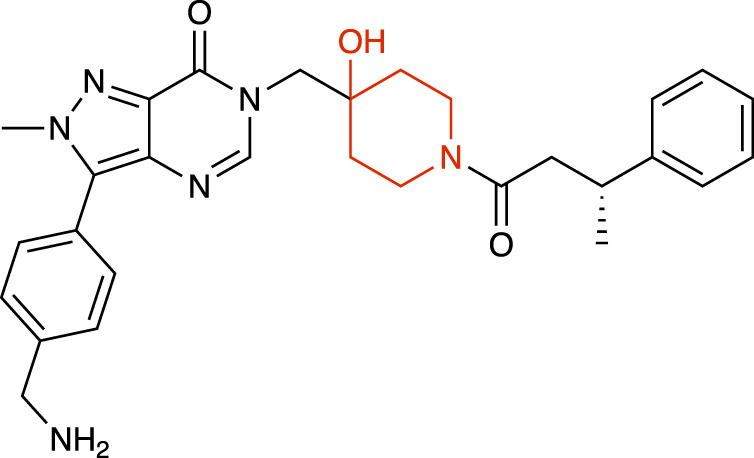	IC_50_ = 6 nM	Colorectal cancer cells	Not found	Gavory et al. (2018); O’Dowd et al. (2018)
4-hydroxypiperidine derivatives FT671 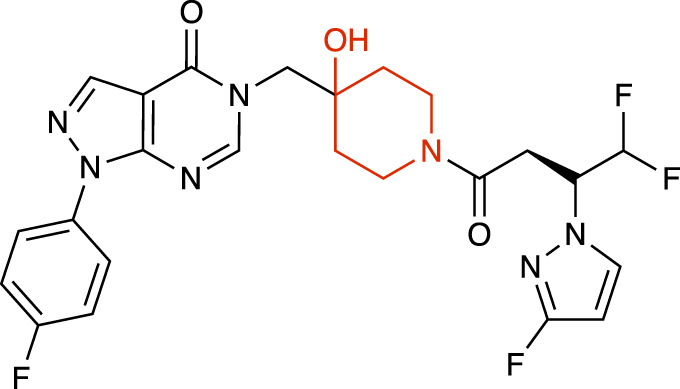	IC_50_ = 52 nM	Colorectal cancer cells, Breast cancer cells	MM xenograft models	Turnbull et al. (2017)
N-benzylpiperidinol derivatives L55 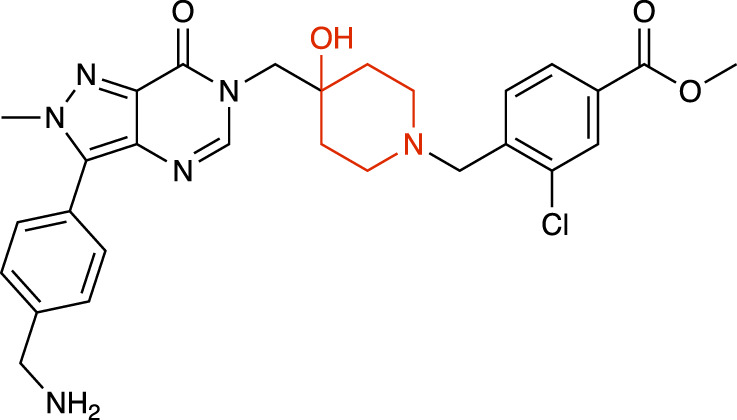	IC_50_ = 40.8 nM	Lymphoblastic leukemia cells, Prostate cancer cells (hormone sensitive)	Not found	Li et al. (2020b)
Thienopyridine derivatives USP7-797 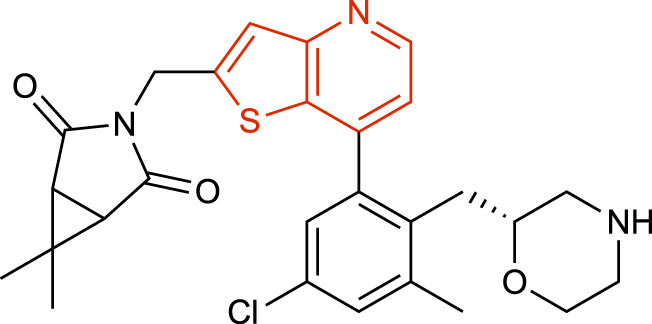	IC_50_ = 0.44 nM	Small cell lung cancer cells (CC_50_ = 450 nM) MM cells (CC_50_ = 89 nM)	Small cell lung cancer and MM xenograft models	Leger et al. (2020); Ohol et al. (2020)

P5091 has been intensively studied and provided the first evidence of *in vivo* anti-tumor activity of USP7i, inhibiting multiple myeloma (MM) growth in a MM1.S xenograft model. It was reported to overcome bortezomib resistance, to trigger synergistic anti-MM activity in combination with Lenalidomide, Suberoylanilide Hydroxamic Acid or Dexamethasone and to synergize with DNA damaging drugs such as etoposide and doxorubicin for killing MM cells *in vitro* (Chauhan et al., 2012). P5091 has also been used in other xenograft models including colorectal cancer (An et al., 2017), glioblastoma (Pan et al., 2021) and esophageal squamous cell carcinoma (Hu et al., 2019), among others.

### Amidotetrahydroacridine derivatives

Two amidotetrahydroacridine inhibitors developed by Hybrigenics, HBX19,818 (Table 1) and HBX28,258, were reported as irreversible USP7i through nucleophilic attack of the catalytic Cys223. The reactive chloro located near the catalytic Cys223 enables the formation of a covalent bond between the carbon atom bearing the chloro and the sulfur atom of Cys223. High-resolution mass spectrometry experiments showed chloride release upon the covalent binding. These active-site-targeting inhibitors did not cross-react with any of the six DUBs tested, and were shown to disrupt cell cycle, leading to G1 cell cycle arrest of colon cancer cells (Reverdy et al., 2012).

### Quinazolin-4-one derivatives

Li and collaborators have synthesized a series of novel quinazolin-4-one derivatives through structure activity relationship studies from a lead molecule, among which C9 and C19 (Table 1) were the most potent USP7i with low micromolar potency (Li et al., 2021). Docking studies predicted that solvent exposure of the side chains of these molecules would result in hydrogen bonds with residues Methionine 407, Phenylalanine 409, Arginine 408, Glutamine 297, Valine 296, Aspartic acid 295 and Tyrosine 465 of USP7, and hydrophobic interaction of the 3-phenylbutanoic acid moiety with the enzyme binding pocket (Li et al., 2021).

### Cyanopyrrolidine derivatives

Structure activity studies of electrophilic peptidomimetic inhibitors were performed and found that inhibitors with a cyanopyrrolidine warhead, such as GNE-3086, GNE-3093 and the most potent compound 11 (Table 1), were able to unexpectedly convert the USP7 active-site Cys223 to dehydroalanine (Bashore et al., 2020). In this reaction, the cyanopyrrolidine warhead promoted a β-elimination reaction of the initial covalent adducts, converting the active-site Cys223 residue to dehydroalanine, abrogating its ability to act as a nucleophile for bond hydrolysis. This irreversibly destroyed the catalytic residue, simultaneously converting the small molecule inhibitor to a non-electrophilic by-product (Bashore et al., 2020).

### Sesquiterpene lactone derivatives

Recently, two sesquiterpene lactones were reported as potential USP7i, Parthenolide (Table 1) and Costunolide, in which the α-methylene- γ -butyrolactone core seems to be important for USP7 inhibition (Li et al., 2020a). Parthenolide activity on USP7 occurred through a Michael addition reaction, centered around the alkylation of a cysteine residue involving the α-methylene- γ -butyrolactone core. However, despite the modification of thirteen cysteines, the catalytic Cys223 did not seem to be modified by Parthenolide. Although Parthenolide preferentially inhibited USP7, it demonstrated partial inhibitory capacity against six other DUBs (USP8, USP21, USP27X, USP30, USP35, and JOSD2). Additionally, it exhibited poor solubility and bioavailability, which are crucial limitations that hinder its potential clinical application (Li et al., 2020a).

### 2-amino-4-ethylpyridin derivatives

Lello and collaborators from Genentech reported GNE-6640 (Table 1) and GNE-6776 allosteric inhibitors as the most selective USP7i reported until then, developed through nuclear magnetic resonance-based screening and structure-based design (Di Lello et al., 2017). Both compounds showed high selectivity when tested against a panel of 36 DUBs. Structural studies revealed that both compounds non-covalently target USP7 12 Å distant from the catalytic Cys223, sterically hindering Ub binding (Kategaya et al., 2017).

### 4-hydroxypiperidine derivatives

Over the last years, a variety of 4-hydroxypiperidine derivatives have been reported as USP7i. Through structure-based design analoguing known hits from the patent literature, Lamberto and collaborators developed XL188 (Table 1) as a non-covalent active-site inhibitor with nanomolar potency and high degree of selectivity relative to other DUBs (Lamberto et al., 2017). XL188 binds to the S4-S5 pocket of USP7, positioned in the thumb-palm cleft and located about 5 Å from the catalytic triad (Cys223, Histidine 464 and Aspartic acid 481), which participates in multiple hydrogen bonds with the four inhibitor heteroatoms. Additionally, the methyl group located at Cα to the phenyl ring was involved in numerous van der Waals interactions that contributed to the inhibitory activity (Lamberto et al., 2017).

The proximity of XL188 to Cys223 suggested that XL188 may serve as the base for a new series of covalent USP7i. Chemical synthesis and biochemical characterization of numerous analogs led to the development of XL177A as a highly potent (sub-nanomolar), selective and irreversible USP7i (Schauer et al., 2020a). XL177A (Table 1) is disclosed to have similar binding sites and binding modes to XL188, however induces additional conformational changes in the USP7 protein dynamics (Schauer et al., 2020a).

O’Dowd and collaborators also identified a series of USP7 inhibitors derivatives of 4-hydroxypiperidine and with a pyrimidine core, from which the allosteric inhibitor ALM34 (Table 1) was the most potent compound, consistently showing IC_50_ values in the single-digit nanomolar range and with high selectivity against other DUBs, including USP47 (Gavory et al., 2018; O’Dowd et al., 2018). Sequence alignment studies indicated that six residues within 5 Å of the inhibitor binding site were different in USP47 compared to USP7, which provided the bases for the inhibitor selectivity (Gavory et al., 2018).

A series of 4-hydroxypiperidine derivatives with a pyrazolo[3,4-d]pyrimidine scaffold has also been reported (Turnbull et al., 2017). Within these series, FT671 (Table 1) was identified as non-covalent inhibitor and FT827 as covalent inhibitor. Through analysis of the inhibitor-USP7 complexes formed, FT671 extended towards the finger’s subdomain through a p-fluorophenyl group, and towards the catalytic center by a 3-fluoropyrazole group that collects against the piperidine group and lies 4.7 Å from the thiol group of Cys223. FT827 was not extended towards fingers subdomain, nonetheless, was elongated towards the catalytic center, supporting the vinylsulfonamide moiety to form a covalent bond with Cys223 (Turnbull et al., 2017).

### N-benzylpiperidinol derivatives

Li and collaborators synthesized 55 piperidinol derivatives, among which L55 exhibited the most potent USP7 inhibitory activity (Table 1) (Li et al., 2020b). X-ray crystallographic studies revealed a new pose for L55 to bind to USP7 between the palm and thumb sub-domains. Upon binding, USP7 Phenylalanine 409 showed a large upshift, thus adding good pi-pi interactions to the pyrazole ring of L55. The (4-aminomethyl) phenyl group stretched to the USP7 surface, participating in hydrogen bond interactions. The pyrazolopyrimidone was buried in USP7 and also participated in multiple hydrogen bond interactions with USP7. L55 showed nanomolar potency and high selectivity against a panel of six DUBs (Li et al., 2020b).

### Thienopyridine derivatives

A new series of thienopyridine derivatives as USP7 allosteric inhibitors with nanomolar potency and high selectivity against a vast panel of DUBs, including USP47, have been reported by RAPT Therapeutics (Ohol et al., 2020). These series possess a novel pharmacophore and bind to a similar pocket as the previously described 4-hydroxypiperidine derivatives, despite belonging to a different chemotype. USP7-797 (Table 1) was one of the most potent compounds with sub-nanomolar potency, oral bioavailability and with an optimal balance of cellular potency and pharmacokinetic properties. It was selected for an *in vivo* MM.1S xenograft study in which it effectively inhibited MM.1S tumor growth and prolonged survival in a dose-dependent manner (Ohol et al., 2020).

## Future perspectives/conclusion

The therapeutic potential of USP7i has been intensively investigated over the last decade. However, albeit promising results have been reported *in vitro* and *in vivo*, no relevant inhibitors have entered clinical trials so far. Emerging evidence suggest that USP7i may be promising to pharmacologically promote anti-tumor immunity. USP7 was proposed as the upstream regulator of programmed death ligand-1 (PD-L1) stabilization in gastric cancer cells contributing to cancer immune resistance and cancer growth. USP7i can attenuate the programmed cell death protein 1 (PD-1)/PD-L1 interaction and sensitize gastric cancer cells to T cell-mediated killing. Therefore, USP7i may be used as novel promoters of the tumor immune response bearing a novel perspective for drug combinations with checkpoint inhibitors, which can broaden the population of patients that respond to PD-1/PD-L1-targeted therapies (Dai et al., 2020; Wang et al., 2021).

Interestingly, it has been reported that, by downregulating the coiled-coil domain containing 6 protein, USP7i can increase the sensitivity of lung neuroendocrine tumors (Morra et al., 2015), hormone-sensitive and androgen-resistant prostate tumors (Morra et al., 2017) and urothelial bladder tumors (Morra et al., 2019) to poly (ADP)-ribose polymerase 1 inhibitors. Additionally, it has been demonstrated that, by increasing the nuclear localization of fructose-1,6-bisphosphatase 1, USP7i increase the sensitivity of pancreatic cancer cells to poly (ADP)-ribose polymerase 1 inhibitors (Cheng et al., 2022).

Notably, USP7 may further play a key role in overcoming resistance to therapy. Using patient-derived xenograft (PDX) human epidermal growth factor receptor 2 positive (HER2+) models it has been demonstrated that the combination of USP7i and trastuzumab synergistically suppressed tumor growth. These provides a rationale for the combination therapy in HER2+ breast cancers that do not respond to anti-HER2 therapy, as well as in other cancers with up-regulated HER2 signaling (Yu et al., 2019). Moreover, in chemosensitive PDX models of small cell lung cancer has been established that overexpression of either *MYCN* or *MYCL* conferred chemoresistance to cisplatin–etoposide. Strikingly, USP7i alone or with cisplatin–etoposide resensitized the chemoresistant models to chemotherapy *in vivo* (Grunblatt et al., 2020).

Despite the design and development of USP7i have proved challenging, there is a considerable body of evidence that USP7i, administered alone or in combination therapies within synergistically pathways, will be a highly valuable new approach that should be pursued for the treatment of cancer.

## Abbreviations

Cys223, Cysteine 223; DUBs, Deubiquitinases; ERα, Estrogen receptor α; HER2+, Human epidermal growth factor receptor 2 positive; HUBL, ubiquitin-like domain; JAMMs, Jab1/Mov34/Mpr1 Pad1 N-terminal+ domain proteases; MDC1, Mediator of DNA damage checkpoint protein 1; MDM2, murine double minute 2; MINDYs, Motif interacting with ubiquitin-containing novel DUB family proteases; MJDs, Machado−Josephin domain proteases; MM, Multiple myeloma; MRN, MRE11-RAD50-NBS1; OTUs, Ovarian tumor proteases; PD-1, Programmed cell death protein 1; PD-L1, Programmed death ligand-1; PDX, Patient-derived xenograft; PHF8, Plant homeodomain finger–containing protein 8; Poly Q, Poly-glutamine; PTEN, Phosphatase and tensin homolog; RNF220, Ring finger protein 220; TRAF, Tumor necrosis factor receptor-associated factor; Ub-AMC, Ubiquitin-7-amido-4-methylcoumarin; Ub, Ubiquitin; UCHs, Ubiquitin C-terminal hydroxylases; UPS, Ubiquitin-proteasome system; USP, Ubiquitin-specific protease; USP7, Ubiquitin-specific protease 7; USP7i, Ubiquitin-specific protease 7 inhibitors; USPs, ubiquitin-specific proteases; ZUP1, zinc finger-containing ubiquitin peptidase 1
